# PrP*^C^* as a Transducer of Physiological and Pathological Signals

**DOI:** 10.3389/fnmol.2021.762918

**Published:** 2021-11-22

**Authors:** Jessica D. Panes, Paulina Saavedra, Benjamin Pineda, Kathleen Escobar, Magdalena E. Cuevas, Gustavo Moraga-Cid, Jorge Fuentealba, Coralia I. Rivas, Human Rezaei, Carola Muñoz-Montesino

**Affiliations:** ^1^Departamento de Fisiología, Facultad de Ciencias Biológicas, Universidad de Concepción, Concepción, Chile; ^2^Departamento de Fisiopatología, Facultad de Ciencias Biológicas, Universidad de Concepción, Concepción, Chile; ^3^Virologie et Immunologie Moléculaires (VIM), Institut National de Recherche pour l’Agriculture, l’Alimentation et l’Environnement (INRAE), Jouy-en-Josas, France; ^4^Université de Versailles Saint-Quentin-en-Yvelines (UVSQ), Versailles, France; ^5^Université Paris-Saclay, Jouy-en-Josas, France

**Keywords:** PrP, Aβ, PrP*^C^* signaling, PrP*^C^* role, PrP*^C^* in CNS, Alzheimer’s disease

## Abstract

After the discovery of prion phenomenon, the physiological role of the cellular prion protein (PrP*^C^*) remained elusive. In the past decades, molecular and cellular analysis has shed some light regarding interactions and functions of PrP*^C^* in health and disease. PrP*^C^*, which is located mainly at the plasma membrane of neuronal cells attached by a glycosylphosphatidylinositol (GPI) anchor, can act as a receptor or transducer from external signaling. Although the precise role of PrP*^C^* remains elusive, a variety of functions have been proposed for this protein, namely, neuronal excitability and viability. Although many issues must be solved to clearly define the role of PrP*^C^*, its connection to the central nervous system (CNS) and to several misfolding-associated diseases makes PrP*^C^* an interesting pharmacological target. In a physiological context, several reports have proposed that PrP*^C^* modulates synaptic transmission, interacting with various proteins, namely, ion pumps, channels, and metabotropic receptors. PrP*^C^* has also been implicated in the pathophysiological cell signaling induced by β-amyloid peptide that leads to synaptic dysfunction in the context of Alzheimer’s disease (AD), as a mediator of Aβ-induced cell toxicity. Additionally, it has been implicated in other proteinopathies as well. In this review, we aimed to analyze the role of PrP*^C^* as a transducer of physiological and pathological signaling.

## Introduction

Prion was first proposed by Stanley Prusiner in 1982 as an infectious protein. This occurred in the context of a group of rare encephalopathies of unknown etiology in sheep and goats, characterized by abnormal trembling termed “scrapie” (Prusiner, 1982). Based on the experiments of ultraviolet irradiation of brain extracts of infected mice, a novel infectious component of low molecular weight was observed which did not depend on canonical transmission by nucleic acids and exhibited replicative and infective capacity (Alper et al., 1967; Griffith, 1967). Later, this infectious particle was isolated and corresponded to a 27–30 kDa protein, which was devoid of nucleic acids, and it was resistant to digestion by proteinase K, which was named “prion” (Bolton et al., 1982; Prusiner, 1982).

Later, several studies revealed that the ability of prion to propagate was related to an abnormally folded variant of prion protein (PrP), which is naturally expressed in mammals (Prusiner, 1982; Collinge, 2001). In this context, normally folded α-helix-enriched cellular prion protein (PrP*^C^*) can be converted into a scrapie protease-resistant form of PrP (PrP*^Sc^*), requiring a cascade of conformational changes to form β-sheet-enriched conformation. Interestingly, PrP*^Sc^* can propagate its own altered conformation using PrP*^C^* as a substrate, in a template replication process (Griffith, 1967; Lansbury, 1994).

PrP*^C^* is highly expressed in different neuronal and astrocytic cells of several central nervous system (CNS) areas, namely, amygdala, cerebellum, hypothalamus, occipital lobe, prefrontal cortex, and spinal cord (Su et al., 2004; Castle and Gill, 2017). It is also moderately or poorly expressed in non-neuronal cells, such as immune system, and endothelial and epithelial cells of colon, uterus, ovary, thyroid, and small intestine (Isaacs et al., 2006; Petit et al., 2013). During embryonic development, the high levels of Prnp messenger RNA (mRNA) have also been found in the CNS and peripheral nervous system (PNS) (Manson et al., 1992; Beringue et al., 2003; Lima et al., 2007; Castle and Gill, 2017).

PrP is a key mediator in several toxicity pathways in some neurodegenerative diseases (NDs), such as Alzheimer’s disease (AD), Parkinson’s disease (PD), Huntington’s disease (HD), and amyotrophic lateral sclerosis (ALS) (Prusiner, 2012; Urrea et al., 2017). In this review, we have focused on summarizing the current knowledge of PrP*^C^* as a sensor and key mediator of physiological significance, its role as a transducer in the amyloid cascade in AD, and its effect on other misfolding-related diseases.

Although PrP*^C^* has been implicated in synapse growth, neural plasticity, and memory, a unified variant of its on-target sites is still unknown. In fact, its mechanisms of action have been the subject of intense research for almost three decades. Despite this, there are fundamental questions that are yet to be solved: (1) What is the biological consequence of the association of PrP*^C^* with normal protein folding process? (2) What is the physiological role of PrP*^C^* interaction with channels? (3) How do PrP species act in association with other misfolded proteins? Based on the current knowledge of the function of PrP*^C^*, we have reviewed the physiological and pathological roles of PrP*^C^* signaling on synaptic function, providing a new angle to the putative role of PrP in health and disease.

## Structure, Processing, and Function of PrP

### Structural Biology of PrP*^C^*

PrP*^C^* is encoded by *PRNP* gene, located in chromosome 20 (in humans) or in chromosome 2 (in mice) (Chesebro et al., 1985; Sparkes et al., 1986). PrP*^C^* is a 210-residue glycoprotein attached to the cell surface by glycosylphosphatidylinositol (GPI) anchor (231–253 residues) (Stahl et al., 1987). Within the plasma membrane, PrP*^C^* is found at lipid rafts (also known as microdomains), enriched in cholesterol and sphingolipids (Simons and Gerl, 2010; Botto et al., 2014; Martellucci et al., 2020). Human PrP genomic cluster also contains the homologous genes *PRND* and *PRNT* of 55 kb, where PRND encodes for a Doppel (Dpl) protein of 179 residues and PRNT encodes three mRNA by alternative splicing, expressed exclusively in the testis (Premzl and Gamulin, 2007). PrP genomic family member also includes Shadoo protein, encoded by *SPRN* gene and located in the human chromosome 10 (Ciric and Rezaei, 2015).

**FIGURE 1:**
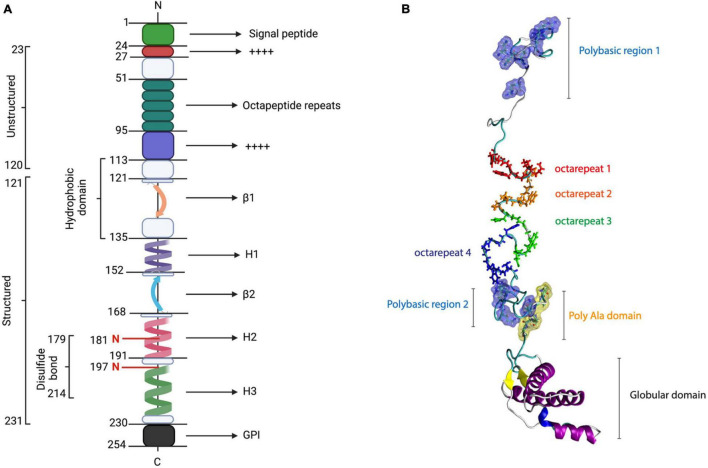
PrP structure. PrP*^C^* consists of 253 amino acids, which include the signal peptide (1–22), five octarepeat regions, a hydrophobic region (113–135), a disulfide bond between its cysteine residues 179 and 214, two *N*-glycosylation sites (residues 187 and 197), and a GPI anchor at its C-terminal. The structured conformation between amino acids 121 and 231 corresponds to a globular domain, which contains two β-sheets and three α-helices. **(A)** Linear representation of PrP sequence [modified from Acevedo-Morantes and Wille (2014)]. **(B)** PrP structure.

The PrP*^C^* first moiety corresponds to a highly positively charged polybasic N-terminal region that is intrinsically disordered and flexible (Beland and Roucou, 2012). Some functions of the N-terminal domain are associated with protein–protein interactions, synaptic transmission, neuroprotection, and Cu^2+^- or Zn^2+^-mediated modulation (Beland and Roucou, 2012; Turnbaugh et al., 2012; Martellucci et al., 2020). Particularly, N-terminal PrP*^C^* contains a signal peptide (1–22 residues) and four functional regions, namely, two positively charged clusters (CC1 and CC2), an octarepeat (OR), and a hydrophobic domain (HD) (Beland and Roucou, 2012; Figure 1).

The PrP*^C^* CC1 domain (23–28 residues) has been associated with myelin homeostasis and PrP*^C^* α-folding stability (Martinez et al., 2015; Kuffer et al., 2016). The OR region (51–91 residues) consists of five octarepeat sequence repeats (PHGGGWGQ), enriched with glycine and histidine (His) residues, which contains several Cu^2+^- and Zn^2+^-binding units (Beland and Roucou, 2012; Wu et al., 2017). Currently, this region has been related to PrP*^C^* endoproteolysis, Cu^2+^ metabolism, and the initial steps of PrP*^C^*–PrP*^Sc^* conversion (Lau et al., 2015).

In contrast, the PrP*^C^* CC2 region (100–109 residues)has been associated with lipid membranes, PrP*^C^* processing, and PrP*^C^* biogenesis (Kim et al., 2001; Wang et al., 2010; Martinez et al., 2015). In the core of the primary structure of PrP*^C^*, it has been described in the HD region (111–130 residues), which seems to have a neuroprotective role against neurotoxicity and myelotoxicity (Beland and Roucou, 2012; Gavin et al., 2020). Interestingly, HD seems to be relevant for the stabilization of PrPC homodimers, contributing to the prevention of prion conversion (Warwicker, 2000; Engelke et al., 2018).

As depicted in Figure 1, the C-terminal globular domain of PrP*^C^* is composed of three α-helix structures, two antiparallel β-sheets, and a GPI anchor (Heske et al., 2004; Sarnataro et al., 2017). Additionally, α-helices 2 and 3 are connected by a disulfide bond between cysteines 179 and 214, contributing to the stability of PrP*^C^*-folded state (Biasini et al., 2012). Another important function that was proposed for the C-terminal domain of PrP*^C^* is its neuroprotective activity against excitotoxicity mediated by Cu^2+^ coordination with N-terminal (Heske et al., 2004; Schilling et al., 2020). The globular domain of PrP is highly conserved among mammals (Salamat et al., 2013) and exhibits high structural similarity with the Dpl, sharing 25% identity (Ciric and Rezaei, 2015). This region is central in the conversion process; however, it allows certain changes, namely, insertions and deletions, in the C-terminal portion of H_2_ without affecting the conversion process (Salamat et al., 2012, 2013; Ciric and Rezaei, 2015; Munoz-Montesino et al., 2016, 2017).

### Sorting and Processing of PrP*^C^*

Since the late 1990s, posttranslational modifications (PTMs) have been recognized as the main regulators of PrP*^C^* biosynthesis. The trafficking of PrP*^C^* precursor protein (253 residues) to the plasma membrane starts with the internalization into the endoplasmic reticulum (ER) by an N-terminal signal peptide (Heller et al., 2003; Chakrabarti et al., 2009; Miranzadeh Mahabadi and Taghibiglou, 2020). After translocation of ER, several PTM occurs to allow PrP*^C^* folding (23–231 residues), namely, C-terminal hydrophobic segment cleavage, C-terminal GPI anchor attachment, and the addition of different patterns of *N*-linked glycosylation (181 and 197 residues in humans, and 180 and 196 residues in mice), which can lead to a diglycosylated, monoglycosylated, or unglycosylated species (Choi, 1992; Zuegg and Gready, 2000; Miranzadeh Mahabadi and Taghibiglou, 2020).

Not all newly synthesized PrP*^C^* is translocated into the plasma membrane. There are two transmembrane (TM) PrP*^C^* topologies that are retained into ER or Golgi for proteasomal degradation, namely, the *N*-transmembrane (NtmPrP*^C^*) and cytosol transmembrane (CtmPrP*^C^*) (Hegde et al., 1998; Sarnataro et al., 2017). There are no precise physiological or pathological functions for NtmPrP*^C^* up to date (Westergard et al., 2007; Miranzadeh Mahabadi and Taghibiglou, 2020). In contrast, CtmPrP*^C^* has been associated with some neurodegenerative pathways, namely, PrP*^Sc^* accumulation, ER stress, cell death, and neurodegeneration (Hegde et al., 1998; Crozet et al., 2008; Gavin et al., 2020).

Later, PrP*^C^* is transported through the Golgi apparatus to the *trans-*Golgi network (TGN), where several PTMs are found to be translocated finally to the plasma membrane, where it remains attached by its GPI anchor (Biasini et al., 2012; Miranzadeh Mahabadi and Taghibiglou, 2020). PrP*^C^* traffics through endocytic recycling compartment, mediated by clathrin-dependent mechanism, where it can be sorted in the plasma membrane for recycling or endolysosomal pathway for degradation (Chakrabarti et al., 2009; Marijanovic et al., 2009; Yim et al., 2015).

The proteolytic processing of PrP*^C^* has been the focus of numerous studies due to physiological or pathological significance of the cleavages, which is still uncertain. Normally, PrP*^C^* can be processed mainly by two proteolytic pathways. First, PrP*^C^* α-cleavage (that occurs at residues 110–111 or 111–112) generates a soluble ∼11 kDa fragment from PrP*^C^* N-terminal domain (N1), as well as a ∼16 kDa fragment from PrP*^C^* C-terminal region which remains attached to the plasma membrane (C1) (Mange et al., 2004; Liang and Kong, 2012). Second, PrP*^C^* β-cleavage releases a longer fragment that remains attached to the membrane (C2) of ∼18 kDa and a ∼9 kDa fragment from PrP*^C^* N-terminal domain (N2) (Biasini et al., 2012; Castle and Gill, 2017).

Although the regulatory role for C1 fragment production is unresolved, it seems that it negatively modulates key steps of PrP conversion process, namely, misfolding, replication, and fibrillization (Westergard et al., 2011; Campbell et al., 2013). Nevertheless, under experimental conditions, C1 lacking the C-terminal portion of H_2_ can be converted into a C1 prion by full-length spontaneous prion harboring the same deletion (Munoz-Montesino et al., 2020). Regarding C2 fragment, data strongly suggest that its accumulation would be a key product of the PrP*^C^* processing in prion replication (Dron et al., 2010). It is likely that C2 represents an important PrP*^Sc^* phenotype-contributing factor during prion disease (Dron et al., 2010).

## Physiological Functions of PrP*^C^*

Although the precise function of PrP*^C^* at the cell surface is not completely understood, some researchers have proposed that it might be important in the nervous system, namely, the formation of synapses, neuronal viability, neuronal excitability, cell motility and neuronal growth, antiapoptotic effect, neurite adhesion, stress sensibility, and calcium homeostasis (Herms et al., 2000; Pantera et al., 2009; Carulla et al., 2011; Park et al., 2015; Wulf et al., 2017; Prado et al., 2020). Additionally, PrP*^C^* has been related to the immune system, namely, T-cell activation, the release of reactive oxygen species (ROS), monocyte maturation, and macrophage phagocytic activity (Isaacs et al., 2006; Miranzadeh Mahabadi and Taghibiglou, 2020). PrP*^C^* also participates in several signaling pathways that regulate innate immunity, namely, Akt, ERK-1/2, and NF-κB (Jeon et al., 2013).

To characterize the physiological function of PrP*^C^*, the initial strategy was to develop PrP*^C^* knockout (KO) mice. The first KOs developed were called Zurich and Npu, both of which did not show marked phenotypes. In both animals, the transmission by prions was completely prevented since the substrate for prion conversion, PrP, was absent (Bueler et al., 1992; Manson et al., 1994). Later, new models, namely, Zurich II, Ngsk, and Rcm0, developed late ataxia due to degeneration of Purkinje neurons (Sakaguchi et al., 1996). In these models, overexpression of Dpl was observed and it would be this protein that causes the death of this type of neurons due to neurotoxicity and not due to the lack of PrP*^C^*. Likewise, it has been established *in vitro* that overexpression of Dpl is toxic only when PrP*^C^* is not expressed; therefore, an interaction between both proteins is suggested to mediate toxicity phenomena (Sakudo et al., 2005).

Likewise, PrP*^C^* modulates growth factor receptor (EGFR) function in regulating cell cycle and growth (Llorens et al., 2013). Another function reported for PrP*^C^* is protection against oxidative stress. It has been determined that in SH-SY5Y neuroblastoma cells in which PrP*^C^* was overexpressed, there was greater resistance to oxidative stress than cells expressing endogenous levels and that this protection would be given by the N-terminal portion of PrP*^C^* (Zeng et al., 2003).

Even though the metal-binding relevance to PrP*^C^* study represents a challenge, a large number of studies support that PrP*^C^* could be involved in copper homeostasis due to its N-terminal unstructured portion. Two main regions are involved in the copper-binding ability of PrP*^C^*: first is the highly conserved octarepeat (OR) region (residues 60–91), where the His residues can bind up to four copper ions with high affinity, and second is the so-called non-OR region (residues 92–111), where two additional His residues are able to bind copper. This non-OR region is contiguous to a hydrophobic portion (residues 112–127) and is thought to be relevant during prion conversion (Giachin et al., 2015). Single His residue mutation in both OR and non-OR regions analyses has supported the idea of the critical role of copper-binding residues, suggesting also its role in regulating the function of PrP*^C^* in neuritogenesis and preserving the functional conformation of the protein, thus contributing to modulate prion conversion propensity (Nguyen et al., 2019). Therefore, copper binding might be relevant to both physiological and pathological roles of PrP*^C^*. Other roles associated with their interaction are endocytosis stimulation and trafficking, antioxidant effect, NMDA receptors modulation, and brain metal homeostasis (Salzano et al., 2019). Metal ion regulation in the CNS has also been related to NDs such as AD and PD (Salzano et al., 2019).

Finally, under physiological conditions in the nervous system, it has been reported that PrP*^C^* is mediating several functions such as cell growth, metal homeostasis, neuritic growth, the formation of lamellipodia, and synaptic transmission (Carulla et al., 2011; Llorens et al., 2013; Legname, 2017; Huang et al., 2018; Nguyen et al., 2019; Prado et al., 2020). The signaling pathways associated with PrP*^C^* neuronal growth-associated functions are achieved by its association with different proteins, such as NCAM and laminin, to promote neurite growth through the activation of Fyn kinase (Schmitt-Ulms et al., 2001; Santuccione et al., 2005). Also, it was determined that PrP*^C^* participates in myelin homeostasis in Schwann cells through interaction with its N-terminal through residues 23–33 with the GPCR 126 receptor on the surface of these cells (Kuffer et al., 2016). The role of PrP*^C^* in neuronal function is further discussed in the subsequent sections.

### Role of PrP*^C^* in Neuronal Function From a Perspective of the Synaptic Transmission

The normal physiological functions and cell behavior of PrP*^C^*, namely, neurite outgrowth, synaptogenesis, synaptic function, and neuroprotection, are not yet well understood. PrP*^C^* has been associated with several intracellular signaling pathways that modulate neuronal signal transduction and it participates in the organization of physiological brain networks, such as neuronal excitability, neuroprotection, neuritogenesis, neurotrophic function, and neuronal plasticity (Linden et al., 2008; Carulla et al., 2015; Castle and Gill, 2017; Linden, 2017). However, to understand how PrP*^C^* can regulate synaptic plasticity by neuronal activity, it is necessary to study the functional interaction of PrP*^C^* with transporters, ion pumps, ion channels, and metabotropic receptors expressed in neuronal cell surface (Table 1). We thus approached PrP*^C^* modulation in two key processes, namely, action potentials (APs) and postsynaptic potentials (PSPs), that coordinate the correct functioning of neuronal performance and the generation of a nerve impulse.

**TABLE 1:** Summary of the main effects of PrP*^C^* in synaptic function.

Binding interaction	Model	Functional role	Proposed mechanism of action	References
VGCC α2δ-1 subunit	Tg PG14 mice (CGNs) Xenopus oocytes and mammalian tsA-201 cells	Glutamatergic neurotransmission Modulation of Ca^2+^ currents	Promotes anterograde trafficking and secretory transport of VGCC channels to the cell membrane Downregulates CaV2.1/β4/α2δ-2 and CaV2.1/β1b/α2δ-1 channels in a GPI-anchoring form	Rutishauser et al., 2009; Senatore et al., 2012; Alvarez-Laviada et al., 2014
Kv4.2 DPP6 subunit	N2a, RK13, and HEK293T cells	Regulation of membrane excitability	Increases peak current amplitudes and the half-inactivation time of A-type K^+^ currents Regulates faster recovery time from steady-state inactivation of Kv4.2 channel	Schmitt-Ulms et al., 2004; Mercer et al., 2013
NMDAR NR1/NR2B subunits	Tga20 knock-in mice (hippocampal neurons) Prnp^0/0^ FVB/N and C57 mice	Neuroprotection Modulation of NMDAR activity	Downregulates NR2D subunits expression and *S*-nitrosylation of NMDAR Reduces glycine affinity, slows inactivation and current amplitudes of NMDAR	Khosravani et al., 2008a; Black et al., 2014; Gasperini et al., 2015; Huang et al., 2018
AMPAR GluA2 and GluA4 subunits	Cultured astrocytes from PrP^–/–^ mice SH-SY5Y and N2a cells Hippocampal neurons from PrP*^C^*-overexpressed mice Tg PG14 and CJD mice	Glutamate-dependent lactate release Zinc uptake Non-affected AMPAR activity Neuronal survival	Regulates the MCT1-associated lactate transport and Na^+^/K^+^ pump astrocytic activity Zinc-sensitive tyrosine phosphatase activity NA Modulates secretory trafficking of AMPAR GluA2 subunit	Kleene et al., 2007; Khosravani et al., 2008b; Watt et al., 2012; Huang et al., 2018; Ghirardini et al., 2020
KARs GluR6/7 and PSD95 subunits	Prnp^0/0^ mice N2a cells Jnk3^0/0^ mice	Neuroprotection against KA toxicity Neuronal survival	Regulates GluR6 and GluR7 mRNA levels Modulates KA-mediated neurotransmission Regulates PSD95/GluR6 complex	Rangel et al., 2007; Carulla et al., 2011, 2015
α7nAChR/STI1 complex	Hippocampal neurons HEK293 cells ZW 13-2 and Zpl 3-4 cell lines from *Prnp*^–/–^ mice	Ca^2+^ homeostasis, neuritogenesis, and neuroprotection	Modulates positively α7nAChR activity PKA activity and ERK1/2 phosphorylation Regulates α7nAchR expression levels	Beraldo et al., 2010; Jeong and Park, 2015

*PrP^C^, cellular prion protein; CGNs, cerebellar granule neurons; CaV, voltage-gated Ca^2+^ channels; DPP6, dipeptidyl aminopeptidase-like protein 6; DPP6, dipeptidyl aminopeptidase-like protein 6; Kv, voltage-dependent K^+^ channels, FVB/N, Friend virus B-type susceptibility-NIH; AMPAR, α-amino-3-hydroxy-5-methyl-4-isoxazole propionic acid receptor; MCT1, astroglial monocarboxylate transporter 1; CJD, Creutzfeldt–Jakob disease; KA, kainate; KARs, kainate receptor; N2a, murine neuroblastoma cell line Neuro2a; JNK3,c-Jun N-terminal kinase 3; PSD-95, postsynaptic density protein 95; α7nAChR, nicotinic acetylcholine receptor; STI1, stress-inducible protein 1; PKA, cAMP-dependent protein kinase 1; ERK1/2, extracellular signal-regulated kinase 1 and 2.*

#### Role of PrP*^C^* in Action Potentials

Collinge et al. (1994) established the role of PrP on neuronal excitability by electrophysiological studies in hippocampal pyramidal neurons and Purkinje cells, from non-transgenic (N-Tg) mice and conditional PrP*^C^*-null mice (Prnp0/0). Interestingly, the histopathological evaluation of Prnp0/0 did not exhibit significant variations with N-Tg mice, but it showed alterations on feedback mechanisms controlling frequency and patterning of neuronal firing, such as input resistance (Rinp), Ca^2+^-activated K^+^ current (IAHP), and afterhyperpolarization (AHP) current (Collinge et al., 1994; Colling et al., 1996; Herms et al., 2001; Mallucci et al., 2002).

One of the main modulators in the generation and shaping of APs is voltage-dependent calcium channels (VGCCs or CaV) (Llinas et al., 1976; Campiglio and Flucher, 2015). Electrophysiological and immunohistochemical studies have shown that PrP*^C^* is able to maintain neuronal excitability at the presynaptic level. This is achieved by stabilization and interaction with α2δ-1 auxiliary subunit of VGCC channels in a GPI anchor-dependent manner (Table 1; Rutishauser et al., 2009; Senatore et al., 2012; Alvarez-Laviada et al., 2014). Furthermore, co-expression of PrP with different Ca^2+^ channel subunits in *Xenopus* oocytes and mammalian tsA-201 cells has shown that PrP is able to modulate the amplitude peak of Ca^2+^ currents of the CaV2.1/β4/α2δ-2 and CaV2.1/β1b/α2δ-1 channels (Alvarez-Laviada et al., 2014). In contrast, in cerebellar granule neurons (CGN) of the transgenic mouse of PrP Tg (PG14), which synthesizes a misfolded mutant variant of PrP (PrPmut) that is partially retained in the ER, it was observed that PrPmut can impair α2δ-1 auxiliary subunit anterograde trafficking, reducing intracellular Ca^2+^ influx and glutamate transmission into the synaptic cleft (Senatore et al., 2012). Furthermore, PrP*^C^* modulates neuronal membrane excitability, synaptic integration of voltage threshold, and the repolarization process of the APs, mediated by their functional interaction with the Kv4.2 (voltage-gated K channels)/DPP6 (dipeptidyl aminopeptidase-like protein 6) complex at the neuronal cell surface (Schmitt-Ulms et al., 2004; Kim et al., 2008; Mercer et al., 2013). Electrophysiological studies in HEK293T cells transiently transfected with the Kv4.2/DPP6 channel complex have shown that PrP*^C^* is able to increase the amplitude peak and depolarizing potential of A-type K^+^ currents, as well as it shifts the activation curve of the Kv4.2 channels to more depolarized potentials in a DPP6-dependent form (Mercer et al., 2013). Further studies are needed to understand the link between PrP*^C^*, its misfolding, and the neuronal activity-dependent signaling pathways during the APs.

#### Role of PrP*^C^* in Postsynaptic Potentials

PrP*^C^* also participates in the regulation of excitatory postsynaptic responses through its functional interaction with ionotropic receptors, namely, *N*-methyl-D-aspartate receptor (NMDARs) (Khosravani et al., 2008a; You et al., 2012), α-amino-3-hydroxy-5-methyl-4-isoxazolepropionic acid receptor (AMPARs) (Watt et al., 2012; Chater and Goda, 2014), kainate receptor (KARs) (Carulla et al., 2011), and α7 nicotinic acetylcholine receptors (α7nAChRs) (Zanata et al., 2002; Beraldo et al., 2010; Roffe et al., 2010; Table 1).

Increasing studies indicate that PrP*^C^* would be a key mediator in the maintenance of glutamatergic synapses, mediated by their interaction with NR1 and NR2 subunits of NMDAR (Khosravani et al., 2008a; You et al., 2012; Gasperini et al., 2015). It was observed that PrP*^C^* ablation induced an overexpression and *S*-nitrosylation of the NR2A and NR2B subunits of NMDAR, altering its kinetic properties. PrP*^C^* ablation induced a slow inactivation of the channel triggering an abnormal increase in neuronal excitability (Gasperini et al., 2015). Meanwhile, overexpression of mouse PrP*^C^* showed decreased activity of NMDAR (Maglio et al., 2004; Khosravani et al., 2008a; Gasperini et al., 2015; Huang et al., 2018). Additionally, recent studies have shown that the neuroprotective effects of PrP*^C^* associated with downregulation of NMDAR would occur in a Cu^2+^−dependent manner (Gasperini et al., 2015; Huang et al., 2018). More studies are needed to establish the interaction sites of PrP*^C^* in the modulation of NMDAR activity.

Regarding AMPA receptors, *in vitro* co-immunoprecipitation studies also revealed interactions with PrP*^C^* (Kleene et al., 2007; Watt et al., 2012; Huang et al., 2018). Interestingly, it has been observed that the increase in the formation of PrP*^C^*/AMPAR complex could exert neuroprotection in a Cu^2+^− and Zn^2+^−dependent manner, as well as AMPA-ergic activity (Watt et al., 2012; Huang et al., 2018). However, PrP*^C^* modulation does not induce significant changes in the amplitude or channel kinetics nor the long-term depression (LTD) maintenance (Khosravani et al., 2008a; Huang et al., 2018). Remarkably, the mutant variant of PrP could exert excitotoxicity mediated by intracellularly retained GluA2 AMPAR subunit (Ghirardini et al., 2020).

It has been postulated that PrP*^C^* has a neuroprotective function in association with KARs against neurotoxicity induced by kainite (KA), which induces neurodegeneration in presynaptic terminals (Carulla et al., 2011). Additionally, *in vivo* and *in vitro* evidence in Prnp0/0 mice indicated that PrP*^C^* can also regulate synaptic transmission and exert neuroprotection against KA toxicity, in a GPI anchoring-dependent manner (Rangel et al., 2007; Carulla et al., 2015). More studies are needed to determine the direct action of PrP*^C^* in channel kinetics and KARs activity, mediated by postsynaptic density protein 95 (PSD95) modulation.

Another postulated mechanism by which PrP*^C^* would exert neuroprotection and promote neuritogenesis is related to its association with α7nAChRs/stress-inducible protein 1 (STI1) complex at the cell membrane (Beraldo et al., 2010). Effectively, in several neuron cell lines, it has been observed that PrP*^C^* can upregulate several neuroprotective pathways, such as autophagic flux cAMP-dependent protein kinase 1 (PKA), and extracellular signal-regulated kinase 1 and 2 (ERK1/2) pathways, in a α7nAChRs-dependent manner (Beraldo et al., 2010; Jeong and Park, 2015).

The overall data suggest that PrP*^C^* would be acting as a new player in the regulation of glutamatergic and cholinergic neurotransmission. However, further research is needed to identify regions involved in the association of ionotropic receptors and PrP*^C^*, as well as the consequences of its disruption in the synaptic neurotransmission in a pathological context.

## Role of PrP*^C^* in Pathology

### PrP*^C^* and Alzheimer

Alzheimer’s disease is a progressive disorder associated with cerebral cortex atrophy and irreversible loss of cortical neurons (Musiek and Schindler, 2013). AD is mainly characterized by an accumulation of amyloid-β (Aβ) plaques and phosphorylated Tau protein neurofibrillary tangles. The major plaque component is Aβ peptide made of 39–43 amino acids, which are derived from the amyloid precursor protein (APP) (Selkoe, 2001; Walsh and Selkoe, 2007). Aβ monomers are not toxic and do not interfere with the synapses, whereas small oligomers and larger aggregates are most likely to be the most toxic species, impairing synaptic plasticity (Legname and Scialo, 2020).

Several studies have related PrP with AD (Kellett and Hooper, 2009); however, the mechanism by which PrP affects the progression of the disease is not clear. Also, there is still controversy regarding whether or not PrP*^C^* is required for Aβ toxicity (Legname and Scialo, 2020). Therefore, we discussed the evidence for interaction of PrP*^C^* and Aβ and its role in mediating Aβ toxicity.

#### Interaction of PrP*^C^* and Aβ

Aβ oligomers (AβOs)-induced neuronal toxicity is thought, at least partly, to be mediated by putative Aβ receptors. Among them, PrP*^C^* has emerged as an important potential receptor, due to its high affinity to the oligomeric form of the peptide (Laurén et al., 2009; Smith et al., 2019; Legname and Scialo, 2020). A cloning cDNA screening from a mouse brain library in order to find a protein that binds to AβOs (Aβ1-42) found that the only high-affinity binding protein was PrP*^C^*, an observation that has been further supported by other studies (Laurén et al., 2009; Corbett et al., 2020). In fact, in a systematic comparison of reported Aβ receptors, only PrP*^C^*, Nogo receptor 1 (NgR1), and leukocyte immunoglobulin-like receptor subfamily member 2 (LilrB2) showed direct binding to synthetic Aβ assemblies. Interestingly, binding with human AD brains-derived soluble AβOs revealed strong affinity only for PrP*^C^*, with a weak affinity for NgR1 and no detectable affinity for LilrB2 (Smith et al., 2019). Therefore, PrP*^C^* is most likely an Aβ-binding receptor.

In contrast to what was observed between PrP*^C^* and AβOs, experiments performed *in vitro* showed low-affinity interactions with Aβ monomers (Chen et al., 2010; Fluharty et al., 2013; Corbett et al., 2020). Solid-phase assays showed that there is neither interaction of monomeric Aβ1-42 with PrP23–231 nor full-length PrP*^C^* (Corbett et al., 2020). However, immunoassay studies have revealed that PrP*^C^* 23–39 and 93–119 can interact with monomeric Aβ1-42 (Kang et al., 2013). Reported sites of interaction between PrP*^C^* and different Aβ species are summarized in Table 2.

**TABLE 2:** Aβ–PrP*^C^* interaction sites.

Binding site of PrP*^C^*	Aβ species	Model	Cellular functions	References
95–110 NA 96–104 NA N-terminus 91–231	AβOs (∼500 kDa) Brain-derived Aβ AβOs (dimers) Aβ protofibrils AβOs (300 and 158 kDa) AβOs (HMW assemblies) AβOs (EC_50_ ∼30 nM)	PrPC-expressing COS-7 cells Prnp^–/–^ and C57Bl6 slices Prnp^–/–^ and APPswe/PSen1ΔE9 slices Aβ-containing AD brain Prnp^–/–^ and C57Bl6 slices Synthetic and Aβ-containing AD brain Tg2576 mice and Aβ-containing AD brains Prnp^–/–^ and C57BL/6J slices	Promotes LTP impairment, cell death, and cognitive impaired	Laurén et al., 2009; Gimbel et al., 2010; Barry et al., 2011; Nicoll et al., 2013; Dohler et al., 2014; Kostylev et al., 2015; Corbett et al., 2020

*PrP^C^, cellular prion protein; AβOs, oligomers of Aβ peptide; LTP, long-term potentiation; Swe, Swedish mutation; PSen1, Presenilin-1, HMW, high-molecular-weight assemblies; AD, Alzheimer’s disease.*

Regarding the binding site in PrP*^C^* for AβOs, it was shown that the unstructured N-terminal domain was relevant for this interaction (Laurén et al., 2009). In fact, when anti-PrP antibodies were used to interfere with the interaction, only 6D11 (which binds to amino acids 93–109 in mouse PrP) blocked the binding between Aβ assemblies and PrP*^C^* with an IC_50_ of 1 nM (Laurén et al., 2009). In addition, the deletion of a similar region (95–105) impaired Aβ binding to PrP*^C^* (Laurén et al., 2009). Another site reported for this binding was the N-terminal basic amino acids 23–27 (KKRPK) in PrP (Legname and Scialo, 2020).

#### PrP*^C^* as a Receptor of Aβ Toxicity

Protein misfolding and aggregation of Aβ peptide are key events in the onset of AD, especially AβOs, due its capacity to associate with the cell membrane and induce excitotoxicity (Puzzo et al., 2017; Cline et al., 2018). The main neurotoxic effects described for AβOs in AD are membrane disruption, synaptic failure, impaired LTP, and memory loss (Lambert et al., 1998; Cline et al., 2018). However, specific binding transducers of AβOs signals that mediate its neurotoxic effects are not yet clearly defined. Several works have postulated different interacting partners for Aβ assemblies in the cell membrane, namely, NMDAR (Rammes et al., 2018), APP (Puzzo et al., 2017), NgR1, nAChR, and PrP*^C^* (Fabiani and Antollini, 2019; Smith et al., 2019; Zhang et al., 2019).

As mentioned earlier, PrP*^C^* has been proposed as a high-affinity physiological receptor for soluble AβOs [see reviews Linden (2017) and Wiatrak et al. (2021)]. At present, in different animal AD models as well as in patients with AD, it has been established that PrP*^C^* could be one of the best specific binding partners for AβOs-mediated inhibition of LTP and cognitive defects in the early stages of AD (Kostylev et al., 2015; Smith and Strittmatter, 2017; Smith et al., 2019; Corbett et al., 2020). With this knowledge, it has been proposed that PrP*^C^* would play an important role in the onset of AD, occurring before clinical symptoms, such as movement and cognitive impairments associated with the late stages of the disease.

**FIGURE 2:**
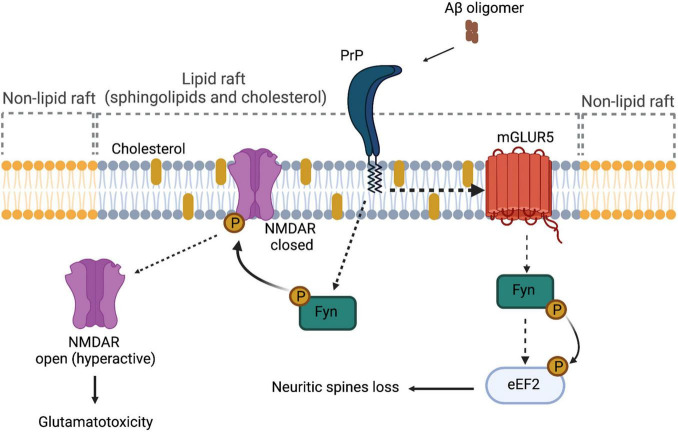
PrP*^C^* as a signal transducer. In the context of Alzheimer’s disease, the interaction between Aβ oligomers and PrP*^C^* affects receptors located on the plasmatic membrane, such as NMDAR and mGluR5. In the case of NMDAR, because of the interaction of Aβ oligomers and PrP*^C^*, the receptor is phosphorylated through Fyn, hyperactivating the channel and causing glutamatotoxicity. In the case of mGluR5, there is a direct interaction between PrP*^C^* and the receptor, causing the activation of Fyn kinase and promoting the phosphorylation of eEF2 and the consequent loss of neuritic spines.

Laurén et al. (2009) have demonstrated that PrP*^C^* is a high-affinity receptor for AβOs, being amino acids 95–110 of PrP*^C^* involved in this interaction (Laurén et al., 2009). Solid-phase and ELISA-like assays showed further associations between AβOs and PrP*^C^* (EC50 ∼30 nM) (Corbett et al., 2020). In cellular and animal models of Aβ toxicity, PrP*^C^* was able to mediate impairment of synaptic plasticity, alteration in calcium transients, and reduction in the levels of synaptophysin (Riek et al., 1996; Laurén et al., 2009; Barry et al., 2011; Peters et al., 2015). Furthermore, these alterations can be rescued using antibodies that block the oligomer-binding site of Aβ in PrP*^C^* (6D11) (Riek et al., 1996; Laurén et al., 2009; Barry et al., 2011; Peters et al., 2015). Regarding the mechanism by which PrP*^C^* exerts its role as a receptor, it has been proposed that AβOs binding to PrP induces activation of Fyn, a Src kinase (SRK), through an undetermined TM partner (Figure 2; Malaga-Trillo and Ochs, 2016). After its activation, fyn phosphorylates NMDA receptor, which becomes transiently over-activated, producing excitotoxicity (Malaga-Trillo and Ochs, 2016). Fyn was already known to be relevant in the pathogenesis of AD, because it performs Tau phosphorylation. Tau is an axonal microtubule-associated protein, and phosphorylated Tau is the main constituent of neurofibrillary tangles in AD, which mediates Aβ toxicity at the post-synapse. The notion that AβO-induced Tau phosphorylation is mediated by PrP*^C^* comes from assays in human and mice brain, as well as analyses in primary neuron cultures, which show that soluble Aβ binds to a PrP*^C^*/Fyn complex and *Prnp* gene deletion uncouples AβOs and the Fyn/tau axis (Larson et al., 2012). Besides Tau phosphorylation, SRKs are able to regulate the stability at the neuronal plasma membrane of several synapse-relevant proteins as adhesion proteins and receptors (e.g., NMDAR, AMPAR, and GABAR) (Malaga-Trillo and Ochs, 2016). Therefore, PrP*^C^*–Fyn interaction might be directly involved in the pathological characteristics of AD. The signal transduction pathway generated by the interaction between AβOs and PrP*^C^* is depicted in Figure 2.

### Role of PrP in Other Neurodegenerative Disorders

Neurodegeneration caused by protein misfolding and aggregation is characterized by progressive neuronal dysfunction associated with deposition of insoluble aggregates from a misfolded protein (Legname and Scialo, 2020). As discussed earlier, in prion diseases, the appearance of PrP*^Sc^* assemblies is involved in this process. In the case of AD, we have mentioned that AβOs, Aβ fibrils and plaques, and Tau tangles appear in the brain of patients with AD and that certain of these species are related to the neurotoxicity and neurodegeneration. In other proteinopathies, amyloids and deposits of other proteins, such as TAR DNA-binding protein 43 (TDP-43), α-synuclein (α-syn), and Tau, are found (Legname and Scialo, 2020; Scialo et al., 2021). In the following sections, how some of these proteins are interconnected with PrP*^C^*, and therefore, the role of PrP in the diseases linked to them are discussed.

#### PrP*^C^* and Tauopathies

Tauopathies are a group of diseases that have in common the deposition of abnormal tau in the nervous system. They comprise AD, Pick’s disease, progressive supranuclear palsy, corticobasal degeneration, and primary age-related tauopathy, among others (Kovacs, 2015). Normal Tau, which is a microtubule-associated protein, plays a role in the stabilization of neuronal microtubules. In pathological conditions, tau undergoes phosphorylation and forms aggregates that are neurotoxic (Avila et al., 2004).

Regarding its relation to PrP*^C^*, *in vitro* and *in vivo* studies have found an association between PrP*^C^* and hyperphosphorylated tau forms, particularly with tau N-terminal region (De Cecco et al., 2020; Legname and Scialo, 2020). Electrophysiological experiments showed that antibodies against PrP*^C^* (6D11, MI-0131) could prevent LTP impairment induced by tau toxicity (Ondrejcak et al., 2018). At present, it has been reported that other antibodies against different epitopes of PrPC (POM 3, 4, 12) are able to impair the uptake of tau amyloid fibrils in mouse neuroblastoma cells (De Cecco et al., 2020). In contrast, it has been described that tau is a transcription regulator for *PRPN* gene in AD models (Lidon et al., 2020), linking both proteins in the progression of tauopathies.

#### PrP*^C^* and α-Synuclein

The misfolding and accumulation of α-synuclein is involved in a group of pathologies known as synucleinopathies, such as PD, dementia with Lewy bodies (LBD), and multiple system atrophy (MSA). For instance, histopathological biomarker detected in patients with PD has been classically associated with abnormal deposits of α-syn, which mainly affects nigral dopaminergic system at the intracellular level, also called Lewy bodies (Kalia and Lang, 2015).

In these diseases, similar to other proteinopathies, fibrillar forms of α-syn spread from one cell to another. One of the mechanisms that this form of α-syn uses to enter cell is clathrin-dependent endocytosis, a process that requires the interaction with the TM protein lymphocyte-activation gene 3 (*LAG3*) (De Cecco and Legname, 2018). Other protein that was reported to be involved in the internalization of α-syn is PrP*^C^* (Aulic et al., 2017). Cells that express PrP*^C^* are able to internalize more amyloid α-syn fibrils compared to cells that do no express it; therefore, PrP*^C^* favors cell-to-cell transmission (Aulic et al., 2017). In contrast, when these cells are infected with prions, α-syn reduces prion replication, especially due to PrP*^C^* α cleavage, producing C1 and N1 that are neuroprotectors (Aulic et al., 2017).

Further analyses agreed on the connection between PrPC and α-syn: overexpression of PrPC in the striatum potentiates neurodegeneration, thereby altering α-syn propagation and toxicity. Electrophysiological and molecular approaches showed that antibodies against PrPC 6D11 could abolish LTP impairment, calcium dyshomeostasis, and cell degeneration induced by α-syn toxicity (Ferreira et al., 2017; Legname and Scialo, 2020).

#### PrP*^C^* and TDP-43

Frontotemporal lobar degeneration (FTLD), a neurodegenerative syndrome in frontal and anterior temporal lobes (Rabinovici and Miller, 2010), and ALS, a motor neuron disorder characterized by degeneration in the upper and lower motor neurons (Prasad et al., 2019), are two distinct diseases that shared a histopathological hallmark: inclusion bodies composed of cytoplasmic deposits of the nuclear TDP-43 protein (Scialo et al., 2021). Under physiological conditions, TDP-43 is a transcriptional repressor that binds to chromosomally integrated TAR DNA. Nevertheless, a hyper-phosphorylated, ubiquitinated, and cleaved form of TDP-43 (pathological TDP-43) is the major disease protein in ubiquitin-positive, tau-, and α-synuclein-negative FTLD and in ALS (Mackenzie et al., 2011; Brauer et al., 2018).

It was observed *in vitro* that TDP-43 fibrils bind to recombinant PrP*^C^*. Also, *in vitro*, it was shown that full-length mouse (Mu)PrP*^C^* as well as human (Hu)PrP*^C^* act as a membrane receptor of TDP-43 in its fibrillar conformation, inducing the formation of intracytoplasmic aggregates and cell death (Scialo et al., 2021). In addition, the overexpression of PrP*^C^* in human and mouse cell lines was directly correlated with the internalization of TDP-43 fibrils. Increased internalization was associated with detrimental consequences in all PrP-overexpressing cell lines (Scialo et al., 2021).

As for other amyloids, treatment with TDP-43 fibrils induced a reduction in the accumulation of the misfolded form of PrP*^C^*, PrP*^Sc^*, in cells chronically infected with prions. Our results expand the list of misfolded proteins whose uptake and detrimental effects are mediated by PrP*^C^*, which encompass almost all pathological amyloids involved in neurodegeneration (Scialo et al., 2021).

## PrP*^C^* in Aging and Other Abnormal Processes

As we mentioned earlier, PrP*^C^* is mostly expressed in the brain. It is especially expressed in the hippocampus and it increases in the aging brain (Williams et al., 2004; Benvegnu et al., 2010). Aging, being the main risk factor for NDs (Wyss-Coray, 2016; Hou et al., 2019), can lead to cognitive impairment, affecting information processing and memory (Hedden and Gabrieli, 2004). Since PrP*^C^* has shown to participate in neuroprotection, metal homeostasis, and most probably as an antioxidant, it has been suggested that it may play a role in aging (Gasperini and Legname, 2014). In fact, in prion diseases, the function of PrP*^C^* is lost due to conversion into PrP*^Sc^* and this event could also be related to the progression of the disease (Gasperini and Legname, 2014). Furthermore, the biochemical properties of PrP*^C^* are altered during aging (Gasperini and Legname, 2014). Even though it is likely that PrP*^C^* is involved in behavior and learning processes during aging, the analyses performed so far in PrP*^C^* KO mice are not conclusive, probably due to differences in mouse models and age (Gasperini and Legname, 2014). Zurich old KO mice exhibit alteration in nest building behavior and decline in associative learning compared to wild-type mice. At molecular level, mice lacking PrP*^C^* showed alterations in cytoskeletal proteins, due to the lower phosphorylation of the neurofilament heavy chain and reduction in B-tubulin III-positive neurons in the hippocampus (Gasperini and Legname, 2014; Schmitz et al., 2014). This might be related to neuronal structure changes due to the absence of PrPC and therefore a cellular explanation to behavioral abnormalities (Gasperini and Legname, 2014; Schmitz et al., 2014). Even though most studies suggest a role for PrP*^C^* in aging, more are still needed to better define this role.

## Conclusion

Although PrP*^C^* studies started from a pathological context, such as prion diseases, in recent years, studies of its functions in physiological terms increased, especially in the nervous system where this protein participates in relevant functions in neural networks, from neurite growth to ion channel association. Despite its important role, it remains a challenge to determine why the lack PrP*^C^* does not show a relevant phenotype and how other proteins might compensate the absence of PrP.

Recently, the role of PrP*^C^* in AD has emerged as crucial, supported by several studies. As this protein does not present TM spans, its interaction with other TM proteins must be key for its role in mediating physiological and pathological phenomena. Since Fyn kinase is a protein involved in both physiological and pathological PrP*^C^*-mediated responses, more studies are needed to understand the differences in the signaling in both processes.

With the discovery that PrP*^C^* is the main receptor for AβOs, more studies are needed to determine whether PrP or other proteins in the pathological pathway might be a target for AD therapy and other NDs.

## Author Contributions

JDP and PS contributed equally to this work in the information search and in the preparation of the manuscript. CMM, JDP, PS, BP, KE, and MEC participated in the figure designed and information search. GMC, JF, HR, CIR, and CMM conducted the manuscript preparation and edited the text.

## Conflict of Interest

The authors declare that the research was conducted in the absence of any commercial or financial relationships that could be construed as a potential conflict of interest.

## Publisher’s Note

All claims expressed in this article are solely those of the authors and do not necessarily represent those of their affiliated organizations, or those of the publisher, the editors and the reviewers. Any product that may be evaluated in this article, or claim that may be made by its manufacturer, is not guaranteed or endorsed by the publisher.
